# COVID-19 data reporting systems in Africa reveal insights for future pandemics

**DOI:** 10.1017/S0950268822001054

**Published:** 2022-06-16

**Authors:** Seth D. Judson, Judith Torimiro, David M. Pigott, Apollo Maima, Ahmed Mostafa, Ahmed Samy, Peter Rabinowitz, Kevin Njabo

**Affiliations:** 1 Department of Medicine, University of Washington, Seattle, WA, USA; 2 Faculty of Medicine and Biomedical Sciences, University of Yaoundé I, Yaoundé, Cameroon; 3 Department of Health Metrics Sciences, University of Washington, Seattle, WA, USA; 4 School of Pharmacy, Maseno University, Kisumu, Kenya; 5 Center of Scientific Excellence for Influenza Viruses, National Research Centre, Giza, Egypt; 6 Reference Laboratory for Veterinary Quality Control on Poultry Production, Animal Health Research Institute, Agricultural Research Center, Giza, Egypt; 7 Immunogenetics, The Pirbright Institute, Surrey, UK; 8 Departments of Environmental and Occupational Health Sciences, Global Health, University of Washington, Seattle, WA, USA; 9 Center for Tropical Research, University of California, Los Angeles, CA, USA

**Keywords:** Africa region, coronavirus, COVID-19, data reporting, pandemic response

## Abstract

Globally, countries have used diverse methods to report data during the COVID-19 pandemic. Using international guidelines and principles of emergency management, we compare national data reporting systems in African countries in order to determine lessons for future pandemics. We analyse COVID-19 reporting practices across 54 African countries through 2020. Reporting systems were diverse and included summaries, press releases, situation reports and online dashboards. These systems were communicated via social media accounts and websites belonging to ministries of health and public health. Data variables from the reports included event detection (cases/deaths/recoveries), risk assessment (demographics/co-morbidities) and response (total tests/hospitalisations). Of countries with reporting systems, 36/53 (67.9%) had recurrent situation reports and/or online dashboards which provided more extensive data. All of these systems reported cases, deaths and recoveries. However, few systems contained risk assessment and response data, with only 5/36 (13.9%) reporting patient co-morbidities and 9/36 (25%) including total hospitalisations. Further evaluation of reporting practices in Cameroon, Egypt, Kenya, Senegal and South Africa as examples from different sub-regions revealed differences in reporting healthcare capacity and preparedness data. Improving the standardisation and accessibility of national data reporting systems could augment research and decision-making, as well as increase public awareness and transparency for national governments.

## Introduction

The COVID-19 pandemic has revealed global differences in pandemic preparedness and response. Previous disparities in detecting, assessing, reporting and responding to international public health emergencies led to the signing of the International Health Regulations in 2005 (IHR 2005) [1]. These regulations contain multiple requirements for all WHO member states, including that they must report any acute public health events that may threaten regional and public health security [1]. However, implementation of the IHR requirements has differed among nations and there has been a lack of coordination between member states [2]. The Joint External Evaluation (JEE) process was adopted in 2016 for voluntary assessment of national capacities to prevent, detect and respond to public health threats; yet there has also been great variation in the participation and performance of nations with the JEE [3]. This variation may include differences in how nations report data during public health events. As we continue to confront the COVID-19 pandemic and prepare for future pandemics, it is important to examine international patterns in data reporting.

Insufficient data reporting by many countries during the COVID-19 pandemic has made them non-compliant with the IHR [4]. Furthermore, variability and instability in global COVID-19 data reporting have created challenges for researchers, public health experts and policymakers, which have led to a call for a standardised approach for data reporting [5]. Additionally, discrepancies in data formatting, interfaces and communication systems create challenges for emergency preparedness and response [6].

Given the unprecedented global scale and rapid progression of the COVID-19 pandemic, countries have had to quickly develop methods for reporting COVID-19 data. Previous comparisons of national COVID-19 data reporting systems have revealed a variety of practices. An analysis of COVID-19 data reporting within the USA showed large variability in data reported between states and highlighted the lack of transparent and standardised COVID-19 data [7]. Internationally, a wide variety of reporting systems has been used by different countries, including a diverse variety of online dashboards which differ in their actionability [8]. Accurate, comprehensive and interoperable national data reporting systems are crucial for online databases that aggregate international data such as the WHO COVID-19 Dashboard and the COVID-19 Dashboard developed by Johns Hopkins University [9, 10].

A prior evaluation of the IHR capacities in the WHO Regional Office for Africa (AFRO) revealed that no country has met all of the IHR requirements and that African countries have limited capacity or no capacity for data reporting based on the JEE [11]. Many African countries have implemented the Integrated Disease Surveillance and Response (IDSR) framework in order to meet IHR requirements. A core function of the IDSR is data reporting, which has been a challenge for many countries [12]. Therefore, examining national data reporting practices among African nations during the COVID-19 pandemic could reveal important insights.

At the beginning of the COVID-19 pandemic, multiple African countries rapidly increased diagnostic and surveillance capacity, enabling further event detection [13]. Yet many initially wondered why there was a lower than expected morbidity and mortality due to COVID-19 in Africa, and whether lower reporting could be contributing to this phenomenon [14]. Comprehensive and reliable reporting of cases, deaths and recoveries are key to understanding this observation. In terms of risk assessment, patient cohorts from North America, Europe and Asia primarily shaped the initial international understanding of epidemiological risk factors for COVID-19 [15–17], and additional data on co-morbidities were needed to identify and address risk factors specific to populations in Africa [18]. Additionally, most African countries quickly enacted non-pharmaceutical interventions such as curfews and lockdowns to mitigate transmission, which came at a cost for healthcare systems and recovery [19]. Data from the outbreak response, such as the number of hospitalisations, tests and therapeutics, are necessary to address health disparities and inequities. For example, the WHO AFRO identified that low testing capacity may have contributed to low detection rates in Africa [20]. Consistent reporting of response data such as testing practices is necessary to confirm such discrepancies. Limited reporting of data can also translate into a lack of meaningful representation in international models and decision-making.

In order to identify and improve practices for data reporting, one must consider the variety of data and reporting systems generated during public health emergencies. The intersection between public health and emergency management is illustrated through the emergency management cycle [21]. A comprehensive model of the cycle includes prevention, preparedness, event detection, risk assessment, response and recovery. Different data are generated at each phase of the cycle, leading to diverse reporting systems and channels through which data are communicated. The IDSR and Rapid Risk Assessment of Acute Public Health Events are WHO technical guidelines that align with the emergency management cycle [22, 23]. During the prevention phase, efforts are focused on mitigating known or unknown threats and may include a variety of surveillance data. This includes indicator-based surveillance (routine collection of predetermined data) and event-based surveillance (*ad hoc* collection of data from acute events) [23]. The preparedness phase involves additional systems planning and training to identify gaps and can include reports on factors such as healthcare capacity [24]. Event detection includes diagnosing and reporting metrics such as cases, deaths and recoveries. The IDSR has a standardised data collection tool for case-based reporting, including variables on region, time, age, sex, occupation, travel history, vaccination status and outcome [22]. Risk assessment involves further evaluating data about the context of the event in order to characterise risk [23]. This includes data from observational studies to address co-morbidities or factors that may increase individual or population-level risk. This may also include genetic sequences of pathogens. The data generated during a response phase vary by the types of pharmaceutical or non-pharmaceutical countermeasures that are employed, including data from clinical trials. Lastly, the recovery phase includes data relating to the resumption of normal or ‘new normal’ operations, including data on long-term sequelae of infection [21, 24]. Data are reported through different systems depending on the phase. National press releases or summaries may convey event detection data, whereas situation reports or online dashboards could also include preparedness, risk assessment and response metrics. These reporting systems can be communicated via different channels, such as websites or social media.

Public health data reporting and information sharing are critical at regional, national and international levels throughout the emergency management cycle. However, there remains little consensus about best practices for data reporting at each of these phases and levels. The IHR requires participating nations to report ‘all available essential information immediately to the appropriate level of health-care response’ [1]. Essential information according to the IHR includes: ‘clinical descriptions, laboratory results, sources and type of risk, numbers of human cases and deaths, conditions affecting the spread of the disease and the health measures employed’ [1]. Meanwhile, the JEE highlights the need for ‘interoperable, interconnected, electronic real-time reporting systems’ and evaluates countries based on whether there are ‘systems for efficient reporting to the FAO, OIE, and WHO’ [25]. The IDSR includes further guidance, recommending that countries use common reporting forms and communication channels [22].

A variety of international sources report COVID-19 data from Africa. The Africa Centers for Disease Control and Prevention (CDC) uses an online dashboard to aggregate COVID-19 cases, deaths and recoveries from official regional collaborating centres and member states [26]. Similarly, the Africa CDC has an online dashboard for national COVID-19 vaccination rates [27]. However, countries also have their own national reporting systems which can provide more detailed data for researchers, decision-makers and the public. These information products can be communicated to recipients through private or public channels. Researchers can quickly access public data to develop predictive models or perform vulnerability assessments. International organisations and policymakers also rely on both private and public data. For instance, the WHO AFRO sourced official COVID-19 data from African nations through emails as well as public websites and social media accounts of member states [28]. Policymakers may make decisions based on public data, and information sharing is a key component of international trust [29]. Public reporting of national data is also important for trust between governments and their citizens. Therefore, assessing publicly available national data can provide important insights into the data reporting practices of nations and identify gaps for mitigating future pandemics.

Publicly shared national data reporting systems may provide additional data variables beyond simply event detection, including additional metrics on preparedness, risk assessment and response. Such national reporting systems may be less accessible and vary in their frequency, content and formatting, so it is important to understand these differences and learn from best practices. As African nations face future emerging infectious diseases and look to strengthen national and continental public health institutions [30], insights from national data reporting during the COVID-19 pandemic will be critical to consider. Therefore, we aimed to compare the types of public national reporting systems and communication channels that African countries used early on in the COVID-19 pandemic.

## Materials and methods

Our first aim was to identify the types of public data reporting systems and channels used by national governments in Africa during the COVID-19 pandemic in 2020. Data reporting systems were defined as information products generated by ministries of health and public health for preparing and responding to the COVID-19 pandemic. Communication channels were the methods used to publicly share these products, such as websites, social media accounts or online repositories.

We examined the 47 countries belonging to the WHO AFRO as well as the seven countries on the African continent belonging to the WHO Eastern Mediterranean region (Djibouti, Egypt, Libya, Morocco, Somalia, Sudan and Tunisia). For these 54 African countries, we searched governmental websites, international repositories, the source materials for aggregated COVID-19 dashboards and social media for official products from national ministries of health and public health containing primary data. Our search therefore excluded countries that did not have official public reporting systems or channels but still share data with organisations such as the WHO and Africa CDC. We compared whether national case counts for all 54 countries were reported by the WHO and Africa CDC dashboards.

We then categorised and compared data reporting systems and channels. Reporting systems were categorised as summaries, press releases, situation reports and dashboards. Communication channels included social media, websites and online repositories. Summaries were defined as text or graphics that provided simple overall counts of event detection data including total cases, deaths and recoveries. Press releases were official statements directed at the press that contained event detection data as well as additional narrative text. Situation reports were defined as daily to monthly published electronic documents, often in PDF format, that had consistent formatting, figures and data beyond total cases/deaths/recoveries. Situation reports provided additional data and details intended for decision-makers. Online dashboards included similar types of data to situation reports but were updated more frequently (often daily or in real-time) and could include interactive figures or graphs intended for a wide audience. Official websites from ministries of health and public health were used to share summaries, press releases, situation reports and dashboards. Social media activity included intermittent postings of summaries, press releases and situation reports on Twitter and Facebook by official accounts from ministries of health and public health.

For the countries that had situation reports and/or online dashboards, we further categorised and compared the variables contained in each report based on the IHR guidelines, which include event detection, risk assessment and response data. Since preparedness data are not included in the IHR guidelines, we did not include these data across all countries. Event detection variables included cases, recoveries and deaths (including among healthcare workers). For risk assessment, we determined whether subnational data were reported as well as sex, age and co-morbidities of cases. Response data variables included total individuals tested, hospitalisations and ICU admissions. Hospital and ICU bed availability as well as oxygen concentrators and ventilators were considered metrics of preparedness. Lastly, we examined regional reporting practices from the five United Nations geoscheme sub-regions (Central, North, East, West and Southern Africa). To provide additional insight into data reporting practices in these sub-regions, we further analysed national reporting in Cameroon, Egypt, Kenya, Senegal and South Africa as examples from each of these sub-regions. These countries were chosen as case studies because of our familiarity with their reporting practices as well as their diversity of data reporting systems and channels. For these countries, we compared their first detected cases, initiation of data reporting, frequency of reporting, data content and accessibility.

## Results

**Fig. 1.**
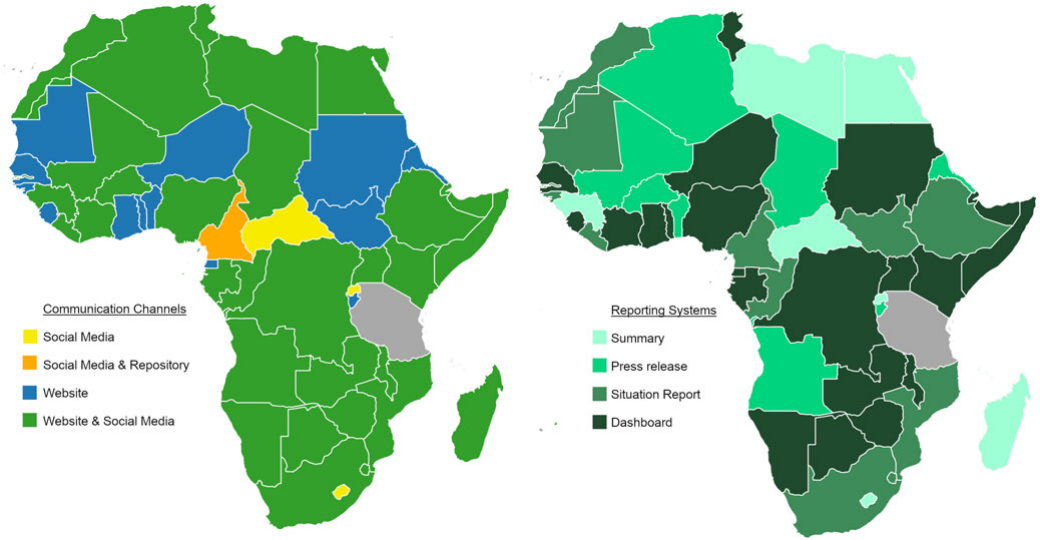
National COVID-19 data reporting systems and communication channels in Africa. African countries are depicted with their identified types of national COVID-19 data reporting systems and communication channels. These public systems and channels contained primary COVID-19 data from national ministries of health and public health. If a country used multiple reporting systems, the more complex system is shown.

All 54 African countries reported diagnosed cases of COVID-19 according to aggregated dashboards [9, 10, 26]. We were able to identify official national COVID-19 reporting systems and channels in 53 of 54 countries (98.1%). Tanzania was the only country without a national public reporting system or communication channel. The national communication channels and systems reporting COVID-19 data in Africa during 2020 are shown in Figure 1. The data variables reported by situation reports and online dashboards are provided in Table 1 with their respective phases of the emergency management cycle. The sources for these systems and channels are provided in Supplementary Table S1. Social media was used by 37/54 (68.5%) of African countries to report official COVID-19 data, and 49/54 (90.7%) used websites.

**Table 1.** Data variables reported by national situation reports and online dashboards in Africa

Country	Event detection					Risk assessment				Response		
	Cases	Deaths	Recovered	HCW cases	HCW deaths	Sub-national	Sex	Co-morbidities	Age	Total Tests	Hospitalised	ICU
Botswana	*	*	*							*		
Cameroon	*	*	*	*	*	*	*	*	*	*	*	
Cape Verde	*	*	*			*			*			
DRC	*	*	*									
Djibouti	*	*	*							*		
Equatorial Guinea	*	*	*	*	*	*	*		*	*	*	
Eswatini	*	*	*	*		*	*		*			
Ethiopia	*	*	*			*				*	*	
Gabon	*	*	*								*	
Gambia	*	*	*			*	*		*	*		
Ghana	*	*	*			*	*					
Guinea-Bissau	*	*	*			*	*	*	*	*		
Ivory Coast	*	*	*			*				*		
Kenya	*	*	*			*	*	*	*	*	*	*
Liberia	*	*	*			*	*			*		
Malawi	*	*	*			*	*		*	*		
Mauritania	*	*	*			*	*					
Morocco	*	*	*			*						
Mozambique	*	*	*			*				*	*	
Namibia	*	*	*			*						
Niger	*	*	*			*						
Nigeria	*	*	*			*	*		*	*		
Rep. of Congo	*	*	*			*	*			*		
Sao Tome and Principe	*	*	*								*	
Senegal	*	*	*			*	*					
Seychelles	*	*	*									
Sierra Leone	*	*	*			*	*		*			
Somalia	*	*	*			*	*					
South Africa	*	*	*	*	*	*	*	*	*	*	*	*
South Sudan	*	*	*	*	*	*				*		
Sudan	*	*	*			*						
Togo	*	*	*			*	*	*	*	*		
Tunisia	*	*	*			*						
Uganda	*	*	*			*	*		*	*	*	*
Zambia	*	*	*			*				*		
Zimbabwe	*	*	*			*	*		*	*		
Frequency	36	36	36	5	4	30	19	5	14	20	9	3

*Variable reported.

Of the countries with reporting systems and channels in 2020, 36/53 (67.9%) had recurrent situation reports and/or updated online dashboards, which contained a variety of data variables (Table 1). All situation reports/dashboards contained event detection data; five countries also included healthcare worker (HCW) cases. In terms of risk assessment, 30/36 (83.3%) had subnational data, 19/36 (52.8%) included patient sex, 14/36 (38.9%) included patient age and 5/36 (13.9%) reported patient co-morbidities. For response data, 20/36 (55.5%) included total number of tests performed and 9/36 (25%) included total hospitalisations.

Comparing sub-regions, Southern Africa had the highest percentage of countries with public situation reports and/or online dashboards, 4/5 (80%), followed by West Africa, 12/16 (75%), Central Africa, 6/9 (66.7%), East Africa, 11/18 (61.1%) and North Africa, 3/6 (50%). We have further examined and compared reporting practices within select countries and their respective sub-regions below.

### Cameroon

On 7 February 2020, the Ministry of Public Health in Cameroon released the first national situation report on COVID-19. Approximately 1 month later, the first case of COVID-19 was officially reported in Cameroon [31]. As the pandemic spread throughout Cameroon, situation reports were published approximately weekly. The content of the reports evolved as the global situation grew and more clinical data became available. The first situation report including hospital bed availability was released on 25 May 2020, while the first report with co-morbidities was published on 31 August 2020. Compared to other countries with COVID-19 reporting systems, Cameroon was one of the few countries that routinely reported preparedness metrics in their situation reports, including data on bed availability and oxygen concentrators. Cameroon was also one of the few countries to report cases among HCWs and diagnostic testing capacity. However, data reporting systems in Cameroon were less publicly accessible compared to other countries. Social media was used to share summaries, and situation reports had to be obtained from the Cameroon Coordination Center for Public Health Emergency and an online humanitarian response repository supported by the United Nations [32]. Five other countries in Central Africa, including the Democratic Republic of the Congo, Equatorial Guinea, Gabon, Republic of the Congo, and Sao Tome and Principe, had online dashboards and/or situation reports.

### Egypt

The first confirmed case of COVID-19 in Africa was announced in Egypt on 14 February 2020 [33]. Early models comparing exported cases and the total number of reported cases by the Ministry of Health in Egypt predicted that the burden of COVID-19 was underreported [34]. The Egyptian Ministry of Health reported summaries of total cases, mortalities and recoveries on a website in Arabic as well as on social media. We were unable to identify public situation reports or online dashboards from Egypt. Therefore, we could not determine the frequency or onset of reporting and did not find preparedness, risk assessment or response data. Only three of six North African countries: Tunisia, Morocco and Sudan, were found to have COVID-19 situation reports or online dashboards.

### Kenya

The first case of COVID-19 in Kenya was confirmed on 12 March 2020, and the first public situation report was published on 1 May 2020. Kenya released frequent situation reports and used an online dashboard to report COVID-19 data. Situation reports were published almost daily during June and July of 2020. Unlike most other countries, Kenya also reported response data, which included hospitalisations, ICU admissions and patients on home-based isolation. Early during the pandemic in Kenya, the distribution and availability of ICU beds and ventilators were a main concern [35]. The majority of countries in East Africa were found to have situation reports and/or online dashboards.

### Senegal

Senegal confirmed its first case of COVID-19 on 2 March 2020 [36], and on 4 March published its first situation report. The Ministry of Health and Social Action in Senegal reported COVID-19 case data using press releases, situation reports and an online dashboard that is updated in real-time. Situation reports were published frequently, roughly weekly at the beginning of the pandemic. Compared to other sub-regions, West Africa had a high percentage of countries with high-quality COVID-19 reporting. One possible reason for this is the experience many of these countries gained in data reporting during the 2013–2016 Ebola virus disease epidemic in West Africa. Timely diagnostics, reporting of cases and contact tracing were critical for both the Ebola virus disease epidemic and the COVID-19 pandemic [37].

### South Africa

On 5 March 2020, the first case of COVID-19 was confirmed in South Africa. Since then, South Africa has identified the most cases of COVID-19 on the African continent, with over 3 965 000 cases identified at the time of this report. The South African COVID-19 Online Resource & News Portal from the Department of Health has frequent updates on COVID-19 statistics. Additionally, the National Institute for Communicable Diseases provided daily hospital surveillance reports since 24 May 2020, which include hospitalisations. One of the first large cohort studies of co-morbidities in COVID-19 patients in Africa came from South Africa [38], and such risk assessment data were also present in their reporting systems. Additional countries in Southern Africa that had online dashboards and/or situation reports included Botswana, Eswatini and Namibia.

## Discussion

Overall, the diversity and differences among COVID-19 reporting systems in African countries reveal international lessons for data reporting during pandemics. Almost all countries reported event detection data, including total cases, deaths and recoveries. Many countries also adopted sophisticated, routine situation reports or online dashboards that could be updated in real-time, which provided additional risk assessment and response data. These additional metrics can be useful for researchers, decision-makers, HCWs and the general public to understand the current status of the pandemic. These data will also be critical for conducting vaccination campaigns, assessing the risk for emerging SARS-CoV-2 variants, and evaluating the long-term sequelae of COVID-19.

Comparing public reporting systems with the IHR, JEE and IDSR can help determine best practices for data reporting during future pandemics. The IHR states that ‘essential information’ to report during a public health emergency should include event detection data (‘cases, deaths, laboratory results’), risk assessment results (‘clinical descriptions, sources and type of risk’) and response data (‘health measures employed’) [1]. While event detection data were widely reported, fewer African countries reported risk assessment data such as co-morbidities and response data such as hospitalisations. These risk assessment and response data are critical for decision-making during public health emergencies. The ‘electronic real-time reporting systems’ recommended by the JEE align with the format of online dashboards, which can contain a variety of data. A strength of online dashboards is that they can be updated in real-time. Situation reports can be communicated via multiple channels and provide historic snapshots for future reference. The tools and guidelines of the IDSR also provide a standardised framework for integrated data reporting systems, and these principles were reflected in the situation reports from African countries.

Multiple African nations went beyond the IHR requirements and reported additional national data relating to healthcare systems and preparedness. For example, Cameroon reported availability of hospital beds and oxygen concentrators. Such data are important for decision-makers when assessing healthcare capacity and allocation of resources. Hospital bed availability has been an important metric in many nations for triggering non-pharmaceutical interventions such as masking and lockdowns. Healthcare capacity data are also important for modellers to predict hospital surges and for HCWs and citizens to assess local risk. Therefore, while the IHR provides a baseline for data reporting, countries need to go beyond these requirements to include healthcare capacity and preparedness data. A main criticism of the IHR is lack of coordination and integration into healthcare systems [2]. Including requirements for healthcare capacity reporting could help integrate the IHR better with healthcare systems. While the IHR and our analysis focused on reporting during current public health events, countries must also consider reporting preparedness data for future pandemics. As the IHR and international data reporting guidelines are revised, it will be important to include best practices for reporting healthcare capacity and preparedness data.

One limitation of our study was that it focused on publicly available reporting systems during 2020, and we may have missed official reporting systems that were less accessible or did not fit our classification paradigm. A review of the COVID-19 response by the WHO AFRO reported that 45 countries submitted situation reports through 2020 [39], and therefore our study likely excluded situation reports that were reported directly to the WHO but were not publicly available online. For instance, no communication channel or reporting system could be found for Tanzania, yet the WHO and Africa CDC reported case data from Tanzania. The absence of a communication channel or reporting system caused many to be alarmed about the Tanzanian government withholding COVID-19 data during the first year of the pandemic [40]. Data reporting systems are essential to keeping the public well-informed and to promote public trust in government, and therefore reporting systems must be accessible. Another limitation is that we were unable to confirm the accuracy of the content of reporting systems. While reporting systems were reviewed through 2020, it is also possible that we may have missed updates or changes to reporting systems and channels.

As regional, national and global entities look to improve pandemic data reporting, they should consider the emergency management cycle and IHR, as well as the diverse systems and channels used by African countries during the COVID-19 pandemic. The IHR, JEE and IDSR provide a foundation for data reporting practices, but there is a need for further clarification of best practices, standardisation and integration. International partnerships between national governments, policymakers, universities and research institutes could help resolve these disparities. Such partnerships, as witnessed during the COVID-19 pandemic, play a key role in the dispersion and comparison of data. As we face future pandemics as an international community, it is essential to reflect on lessons from the COVID-19 pandemic in order to develop and improve data reporting systems.

## Data Availability

The data that support the findings of this study are available within the article and its Supplementary materials.

## Supplementary material

For supplementary material accompanying this paper visit https://doi.org/10.1017/S0950268822001054. click here to view supplementary material
