# Supplementary material for: COVID-19 data reporting systems in Africa reveal insights for future pandemics
Source: Epidemiol Infect. 2022 Jun 16;150:e119. doi: 10.1017/S0950268822001054 (PMC9237488; doi:10.1017/S0950268822001054)
Supplement: Supplementary file 1 [file S0950268822001054sup001.docx]

Epidemiology and Infection

**COVID-19 data reporting systems in Africa reveal insights for future pandemics**

Seth D. Judson, Judith Torimiro, David M. Pigott, Apollo Maima, Ahmed Mostafa, Ahmed Samy, Peter Rabinowitz, Kevin Njabo

Supplementary Material

| Supplementary Table S1. National sources for COVID-19 Data in Africa | | |
| --- | --- | --- |
| **Country** | **Reporting System/**  **Channel*** | **Source** |
| **Algeria** | social media | <https://www.facebook.com/pg/SanteDZA/posts/> |
|  | website | http://www.sante.gov.dz |
|  | press release | http://www.sante.gov.dz/coronavirus/coronavirus-2019/82-documentation/531-point-de-situation.html |
| **Angola** | website  press release | http://www.covid19.gov.ao/ |
|  | social media | <https://twitter.com/COVID19GovAo>https://www.facebook.com/COVID19GovAo |
| **Benin** | press release | [https://sante.gouv.bj//CORONAVIRUS-COMMUNIQUE-DU-MINISTRE-DE-LA-SANTE](https://sante.gouv.bj/CORONAVIRUS-COMMUNIQUE-DU-MINISTRE-DE-LA-SANTE) |
| **Botswana** | dashboard | <https://covid19portal.gov.bw/data-dashboard?v=202005211151> |
|  | social media | https://twitter.com/mohwbotswana?lang=en |
| **Burkina Faso** | social media | https://twitter.com/SANTE_BF? |
|  | press release | https://www.sig.gov.bf/actualites/communiques? |
| **Burundi** | press release | <http://minisante.bi/?p=809> |
| **Cameroon** | social media | <https://twitter.com/DrManaouda> |
|  | situation report | <https://www.humanitarianresponse.info/en/op%C3%A9rations/cameroon/documents/document-type/situation-report/themes/covid-19?page=1> |
| **Cape Verde** | dashboard | <https://covid19.cv/> |
|  | social media | https://www.facebook.com/ministeriodasaude.cv/ |
| **Central African Republic** | social media | https://twitter.com/mspcentrafrique?lang=en https://www.facebook.com/RCAMSP/ |
| **Chad** | press release | http://sante-tchad.org/communique-de-presse/ |
|  | social media | https://www.facebook.com/ministeresantetchad/ |
| **Comoros** | press release | <https://stopcoronavirus.km/actualit%C3%A9s/2020/08/12/communiqu%C3%A9-n%C2%B0041-du-09-aout-au-11-aout-2020/> |
|  | social media | https://www.facebook.com/Minist%C3%A8re-de-la-Sant%C3%A9-Union-des-Comores-320950001695355/ |
| **Democratic Republic of Congo** | social media | [https://twitter.com/cmr_covid19_rdc](https://cmr-covid19.cd/rdc-cases) |
|  | dashboard | https://cmr-covid19.cd/rdc-cases https://www.stopcoronavirusrdc.info/ |
| **Djibouti** | situation report  dashboard | <https://sante.gouv.dj/> |
|  | social media | https://twitter.com/MinSantedj |
| **Egypt** | social media | <https://twitter.com/mohpegypt/status/1323385676646305796>  https://www.facebook.com/idsc.gov.eg/ |
|  | website | <https://www.care.gov.eg/EgyptCare/Index.aspx;> |
| **Equatorial Guinea** | dashboard | <https://guineasalud.org/estadisticas/> |
| **Eritrea** | press release | <https://shabait.com/2020/12/24/announcement-from-the-ministry-of-health-95/> |
| **Eswatini** | situation report | <http://www.gov.sz/index.php/covid-19-corona-virus/situational-analysis> |
|  | social media | https://twitter.com/eswatinigovern1?lang=en |
| **Ethiopia** | social media | <https://twitter.com/FMoHealth?ref_src=twsrc%5Egoogle%7Ctwcamp%5Eserp%7Ctwgr%5Eauthor> |
|  | website | [https://www.ephi.gov.et/](http://www.moh.gov.et/ejcc/en) |
|  | situation report | <http://www.moh.gov.et/ejcc/en> |
| **Gabon** | situation report  dashboard | <https://infocovid.ga/> |
|  | social media | https://twitter.com/Covid19GOUVGA  https://www.facebook.com/Covid19GOUVGA/ |
| **Gambia** | situation report | <http://www.moh.gov.gm/covid-19-report/> |
|  | social media | https://www.facebook.com/MohCovid19GMB/  https://twitter.com/MoHCovid19GMB |
| **Ghana** | dashboard | <https://www.ghanahealthservice.org/covid19/> |
| **Guinea** | website | [https://anss-guinee.org/welcome#](https://anss-guinee.org/welcome) [https://sante.gov.gn/#](https://sante.gov.gn/) |
|  | social media | https://twitter.com/anss_guinee |
| **Guinea-Bissau** | website  situation report | <https://accovid.com/index.php/2020/10/26/boletim_informativo_semanal_covid_nr_09_2020/> |
| **Ivory Coast** | dashboard | <https://coronavirustracking.ci/> |
|  | social media | https://twitter.com/gouvci?lang=en |
| **Kenya** | press release | <http://www.health.go.ke/press-releases/> |
|  | situation report | <https://www.health.go.ke/#1591180376422-52af4c1e-256b> |
|  | dashboard | <https://covid19.health.go.ke/> |
|  | social media | https://twitter.com/gouvci?lang=en  https://www.facebook.com/MinstryofHealthKE/ |
| **Lesotho** | social media | <https://twitter.com/nacosec?lang=en> |
| **Liberia** | social media | [https://www.facebook.com/pages/category/Government-Organization/National-Public-Health-Institute-of-Liberia-NPHIL-164280647325112/](https://www.facebook.com/National-Public-Health-Institute-of-Liberia-NPHIL-164280647325112/?__tn__=C-R&eid=ARCCIP_ufIbjX6EYZ8bsFyAU9UoK_yVGQ72UZ1yYpK271QHkIFOfhUkc9v-b6u5w76okviQdSSZR2xn5&hc_ref=ARRntmDZQA-SrRwAuauTc_oBZdX2gwj1PYqnUKXt--PkHaJiyurnKI6k6r9fDPLzWRc) |
|  | situation report | <http://moh.gov.lr/documents/reports/covid-19/2020/covid-19-sitrep-vol-17/> |
| **Libya** | social media | <https://www.facebook.com/NCDC.LY/> |
|  | website | <https://ncdc.org.ly/Ar/> |
| **Madagascar** | social media | <https://www.facebook.com/minsanp/?ref=page_internal> |
|  | website | <http://cco-covid19.gov.mg/fr/accueil> |
| **Malawi** | situation report  dashboard | <https://covid19.health.gov.mw/> |
|  | social media | https://twitter.com/health_malawi?lang=en  https://www.facebook.com/malawimoh/ |
| **Mali** | social media | <https://www.facebook.com/msdsmali1/posts/2832790576950442> |
|  | press release | http://www.sante.gov.ml/ |
| **Mauritania** | website  situation report | <https://www.sante.gov.mr/?p=4249> |
| **Mauritius** | social media | <https://www.facebook.com/coronavirusmoris/> |
|  | press release | <https://health.govmu.org/Pages/readmorenews.aspx?n=Rehabilitation-works-of-Cavendish-Bridge-to-kick-off-in-May-2020.aspx> |
| **Morocco** | website | <http://www.covidmaroc.ma/Pages/AccueilAR.aspx> |
|  | situation report | http://www.covidmaroc.ma/Pages/LESINFOAR.aspx |
|  | social media | https://twitter.com/ministere_sante?lang=en |
| **Mozambique** | social media | https://www.facebook.com/MISAUMOCAMBIQUE/ |
|  | situation report | <https://covid19.ins.gov.mz/dados-e-estatisticas/> |
| **Namibia** | social media | <https://www.facebook.com/MoHSSNamibia/> |
|  | press release | <https://mhss.gov.na/> |
|  | dashboard | <https://namibia-covid-19-data-hub-nsa-online.hub.arcgis.com/> |
| **Niger** | dashboard | <https://coronavirus.ne/carte-interactive/> |
| **Nigeria** | situation report | <https://ncdc.gov.ng/diseases/sitreps/?cat=14&name=An%20update%20of%20COVID-19%20outbreak%20in%20Nigeria> |
|  | dashboard | <https://covid19.ncdc.gov.ng/> |
|  | social media | https://twitter.com/ncdcgov?lang=en |
| **Republic of the Congo** | social media | <https://twitter.com/MSPPFIFD_cg> |
|  | situation report | <http://sante.gouv.cg/category/documentations/> |
| **Rwanda** | social media | <https://twitter.com/rwandahealth?lang=en> |
| **Sao Tome and Principe** | dashboard | https://covid.ms.gov.st/st/ |
|  | social media  situation report | https://www.facebook.com/MSaudeSTeP/ |
| **Senegal** | situation report | <http://www.sante.gouv.sn/Actualites/coronavirus-communiqu%C3%A9-de-presse-n%C2%B0246-du-02-novembre-2020-du-minist%C3%A8re-de-la-sant%C3%A9-et-de> |
|  | dashboard | <https://sante.sec.gouv.sn/> |
| **Seychelles** | dashboard | <http://www.health.gov.sc/index.php/covid-19/covid-19-in-seychelles/> |
| **Sierra Leone** | situation report  dashboard | <http://dhse.gov.sl/> |
| **Somalia** | dashboard | <https://moh.gov.so/en/covid19/> |
|  | social media | https://twitter.com/MoH_Somalia?ref_src=twsrc%5Egoogle%7Ctwcamp%5Eserp%7Ctwgr%5Eauthor https://www.facebook.com/MoHSomalia/ |
| **South Africa** | situation report | <https://sacoronavirus.co.za/category/press-releases-and-notices/>https://www.nicd.ac.za/diseases-a-z-index/covid-19/surveillance-reports |
|  | social media | https://www.facebook.com/HealthZA/  https://twitter.com/HealthZA?ref_src=twsrc%5Egoogle%7Ctwcamp%5Eserp%7Ctwgr%5Eauthor |
| **South Sudan** | situation report | <http://moh.gov.ss/weekly_updates.php> |
| **Sudan** | situation report  dashboard | <http://www.sho.gov.sd/corona/> |
|  | social media | https://twitter.com/sdn_health?lang=en |
| **Tanzania** | none identified |  |
| **Togo** | dashboard | <https://covid19.gouv.tg/graph-evolution/> |
| **Tunisia** | dashboard | <https://covid-19.tn/fr/tableau-de-bord/> |
|  | social media | https://www.facebook.com/santetunisie.rns.tn/ |
| **Uganda** | dashboard | https://www.health.go.ug/covid/ https://covid19.gou.go.ug/ |
|  | social media | https://twitter.com/MinofHealthUG?ref_src=twsrc%5Egoogle%7Ctwcamp%5Eserp%7Ctwgr%5Eauthor [https://www.facebook.com/minofhealthUG](https://www.facebook.com/minofhealthUG/)/ |
| **Zambia** | dashboard | https://www.moh.gov.zm/ |
|  | social media | https://www.facebook.com/mohzambia/ https://twitter.com/mohzambia?ref_src=twsrc%5Egoogle%7Ctwcamp%5Eserp%7Ctwgr%5Eautho |
| **Zimbabwe** | situation report | <http://www.mohcc.gov.zw/index.php?option=com_phocadownload&view=category&id=15&Itemid=741> |
|  | social media | https://twitter.com/MoHCCZim?ref_src=twsrc%5Egoogle%7Ctwcamp%5Eserp%7Ctwgr%5Eauthor |

*Websites and social media contain summaries unless a situation report is listed with the same source
